# Unraveling Nature’s Pharmacy: Transforming Medicinal Plants into Modern Therapeutic Agents

**DOI:** 10.3390/pharmaceutics17060754

**Published:** 2025-06-07

**Authors:** Natalia Vaou, Chrysoula (Chrysa) Voidarou, Georgios Rozos, Chrysa Saldari, Elisavet Stavropoulou, Georgia Vrioni, Athanasios Tsakris

**Affiliations:** 1 Department of Microbiology, Medical School, National and Kapodistrian University of Athens, 11527 Athens, Greece; chrysasaldari@gmail.com (C.S.); elisabeth.stavropoulou@gmail.com (E.S.); gvrioni@med.uoa.gr (G.V.); atsakris@med.uoa.gr (A.T.); 2 Laboratory of Animal Health, Hygiene and Food Quality, School of Agriculture, University of Ioannina, 47100 Arta, Greece; giorgosrozos286@gmail.com; 3 Laboratory of Animal Husbandry and Nutrition, Department of Agriculture, University of Western Macedonia, 53100 Florina, Greece; 4 Laboratory of Hygiene and Environmental Protection, Department of Medicine, Democritus University of Thrace, Dragana, 68100 Alexandroupolis, Greece

**Keywords:** medicinal plants, traditional medicine, secondary metabolites, drug discovery, drug development, cutting-edge techniques, omics technologies

## Abstract

Natural products (NPs) serve as a crucial source for leading bioactive compounds in drug discovery research. Numerous drugs currently utilized as therapeutic agents have been derived from natural origins, with medicinal plant (MP) sources being particularly significant. Despite the advancement of synthetic chemistry, the importance of NPs persists due to their distinct chemical structures and varied biological activities. Moreover, recent advancements in technology have significantly aided in overcoming challenges, primarily due to inherent complexity. This review explores the potential of NPs in the process of drug discovery and development, placing emphasis on the blend of traditional knowledge with modern drug discovery techniques. A brief history of the development of NP drug discovery and examples of significant NPs developed in recent decades are also provided. The focus is on the various methods employed in authentication, selection, extraction/isolation, and bioactivity screening through the application of modern drug-development principles for NPs. Several cutting-edge techniques, such as genetic engineering, metabolic engineering, plant cell culture and synthetic biology utilizing “omics” technologies and computational methodologies enhancing research in NP drug discovery, are also highlighted. There are several problems and inherent challenges regarding NP drug discovery that need to be overcome. Despite the challenges that exist, NPs will be crucial for the future development of new therapeutic drugs, and it is expected that continuous research and the effective utilization of new approaches will further enhance drug discovery efforts.

## 1. Introduction

For thousands of years, medicinal and aromatic plants have served as a natural source for treatment and healthcare remedies, differing across various cultures and territories and forming, thus, what modern science refers to as ethnopharmacology [1]. Evidence suggests that herbs were used for medicinal purposes even from paleolithic and neolithic times [2]. In historical times, the Chinese book Shannong’s Herbal Atlas, written from 221 BC to 220 AD, contains information on the pharmacological activity of various herbs and plants as well as their toxicity [3]. In classical Greek antiquity, the legendary figures of Theophrastus of Eresos (370-287 BC), Hippocrates of Kos (ca460-370 BC), Galen (129-200 AD) and Pedanios Dioscurides (1st century AD) laid the scientific, although entirely empirical, foundation of pharmacology [4]. Arab scholars and doctors, such as Al Tabbani, Al Razi and the most famous of all, Avicenna (980-1037), not only received Greek knowledge of medicinal plants but returned it significantly enriched to medieval Europe [5].

The ethnopharmacological applications of plants have been a crucial source of leading bioactive compounds during the initial phases of drug discovery and development [6]. Traditional knowledge of the therapeutic uses of MP materials has significantly contributed to the extraction of individual chemical bioactive compounds and their integration into modern medicine. Various fields of study and a range of investigative techniques have been incorporated into the search for drugs derived from MPs. The extensive use of plants as medicinal agents has necessitated the extraction of natural products (NPs) or secondary metabolites (SMs), which began with the early 19th-century (1803) isolation of morphine from Papaver somniferum by Friedrich Sertuuner [7]. The identification of early pharmaceuticals, such as digitoxin by Sydney Smith in 1930 [8], cocaine by Albert Nieman in 1859 [9], pilocarpine, codeine and quinine [10] derived from MPs represented a remarkable milestone in medical science. Additionally, beyond these historical discoveries, numerous other bioactive compounds sourced from MPs have been identified more recently. These substances have undergone thorough research and development, leading to their commercialization as pharmaceutical products [11]. Beyond the identification of new chemical entities for therapeutic purposes, NPs play a critical role as potential leading compounds for crafting new and more effective medicines via structural modifications. Although NPs contain a vast array of intricate chemical structures, they tend to exhibit superior biological compatibility and drug-like properties compared to those derived from entirely synthetic origins. As a result, bioactive compounds of natural origin are likely to serve as better candidates for further pharmaceutical development [12].

Ongoing research underscores nature’s role as an indispensable source of bioactive compounds that drive the discovery of novel therapies. To standardize natural product–based treatments and pinpoint analytical marker biomolecules, researchers have adopted a variety of drug-development strategies [6]. Pharmaceuticals derived from medicinal plants are produced by applying biotechnological techniques to optimize lead compounds sourced from natural products, thereby equipping the healthcare industry with powerful agents against serious diseases [13]. Compared to traditional discovery methods, these biotechnologically engineered, plant-derived drugs offer greater efficiency, cost effectiveness, and safety [14].

Natural products, typically categorized into three main classes (phenolics, alkaloids, and terpenoids), exhibit a wide range of pharmacological properties, such as antimicrobial, antioxidant, anti-inflammatory and anticancer, among others [15,16]. Therefore, NPs are utilized for treating and/or preventing numerous common diseases in humans, such as infections, cancers, disorders related to the respiratory, digestive and cardiovascular systems, diabetes and inflammatory conditions, as well as conditions requiring immunomodulation, anticoagulant and antioxidant treatments. In response to the widespread emergence of antibiotic-resistant bacteria and pandemics, the research and application of NPs have attracted substantial attention within the infectious disease community [17]. The demonstrated antimicrobial efficacy of MP extracts and their derivatives has opened avenues for investigating new and potent treatments against multidrug-resistant pathogens. Additionally, NPs could be used as potential new leads for drug development targeting severe acute respiratory syndrome coronavirus 2 (SARS-CoV-2) or novel coronavirus (COVID-19) [18].

Natural products are also crucial for the biodefense and environmental adaptation of MPs. NPs are chemical signals that mediate MPs’ interactions with symbiotic microorganisms, as well as attract pollinators and seed dispersers [19]. 

Natural product extraction strategies have evolved over the years, taking into account factors such as the characteristics of the source material, solubility, molecular structure, and the chemical nature of both the bioactive compounds and the organic solvents employed [19,20]. Traditional extraction techniques face some drawbacks, such as lengthy times for effective extraction, along with lower overall efficiency when analyzing the relative yields as well as lower specificity of the extracted bioactive compounds. These methods often require substantial amounts of organic solvents, which can be detrimental to the environment and costly to purchase and discard. However, recycled extraction or mainly supercritical CO_2_ are utilized in many large-scale processes. To address these shortcomings of conventional methods, a variety of advanced techniques have been created, alongside the introduction of innovative solvents that are more environmentally friendly, less toxic, economically viable, and more selective [21]. Advanced extraction methods primarily involve assisted extraction techniques using ultrasound, pressurized liquids, microwaves, and pulsed electric fields, with the core objective of damaging cell walls to improve mass transfer and ensure effective mixing by exposing cytoplasmic contents to the solvent [22]. Despite the potential of these green technologies, there are still hurdles in enhancing their efficiency, ensuring cost effectiveness, and applying a comprehensive life cycle perspective to waste management [23,24].

The increasing frequency of global natural changes poses a significant threat to the growth of MPs, resulting in unstable yields of active compounds. Furthermore, wild harvesting, which is a major source of NPs, is leading to genetic diversity loss and habitat destruction. Therefore, modern medicine urgently requires advanced technologies and strategies to ensure the quality and security of MPs while enhancing the yield of NPs to support sustainable and eco-friendly development. Among these effective, commonly employed biosynthetic systems and methods for efficient large-scale production of bioactive compounds are genetic engineering, plant cell culture engineering, metabolic engineering, and synthetic biology supported by various “omic” technologies [25,26,27].

Environmental conditions greatly dictate the synthesis and eventual accumulation of NPs, and a change in any factor leads to disturbances in the biosynthesis of NPs [28]. Key environmental factors influencing the synthesis of NPs include photoperiod, light intensity, water availability, temperature, soil composition, and biotic interactions [29]. For example, research has indicated that higher light intensity can influence the NPs, such as flavonoids, alkaloids and terpenoids [30]. Biotic factors, including herbivore grazing or pathogen attacks, can stimulate the production of defense-related NPs as a response to stress [31]. Moreover, due to climate change, the adverse effects of extreme temperatures, salinity and drought highlight the importance of plant growth and productivity [32].

The development of NP therapeutics faces considerable technical and financial challenges, including formulation processes, quality and safety control, therapeutic effectiveness, marketing and regulatory problems. To address these challenges, regulatory bodies are working to incorporate these medicines into the regulatory framework. Regulatory aspects surrounding NP medicines should be a significant global concern, as every country poses its own unique regulatory hurdles and frameworks that can greatly influence the creation, promotion and distribution of plant NPs. Regulatory scientists around the globe are increasingly supporting scientific regulations of NPs to ensure the safety and efficacy recognizing incidents of clinically significant toxicity associated with various MP products [33].

Despite the clear achievements in MP drug discovery, upcoming efforts encounter numerous challenges. Faster and more effective methodologies for plant collection, bioassay screening, efficient compound isolation, and NP discovery and development need to be utilized [34]. Moreover, the design, assessment and execution of clinically relevant, high-throughput bioassays pose significant challenges for all drug discovery programs [35]. NPs are generally isolated in limited quantities, insufficient for lead optimization, lead development and clinical trials [36]. Furthermore, authenticating medicinal products (MPs) is particularly challenging owing to the inherent chemical complexity and diversity of natural products (NPs) [37].

Although there is a wealth of experience with the use of MPs in traditional medicine, conducting scientific research and identifying the active compounds within these MPs can uncover new therapeutic advantages and facilitate the creation of new NPs in the future. However, advancing any promising leading bioactive compound necessitates large-scale extraction or biotechnological production to make these bioactive compounds clinically applicable [38]. In the future, some effective resources for NP drug development will include advanced methods supported by sophisticated instruments, well-known biological assays, and systematic evaluations intended for clinical trials [39].

The main objective of this narrative review is to present cutting-edge methodologies employed for enhancing NPs from MPs, detailing the processes. Bridging traditional knowledge with modern scientific approaches is also highlighted for optimizing the potential of NPs in drug discovery. Chemical diversity, complex network of biosynthetic pathways, pharmacological properties and the effects of environmental factors on the production of NPs are critically analyzed. The establishment of effective regulating frameworks, problems, challenges and future prospects for the discovery of drugs from NPs are also discussed.

## 2. Methodology

A thorough literature search was carried out to identify any studies related to the MP-derived NPs utilized in drug discovery and development. To conduct this narrative review, two major databases (PubMed/Medline and Scopus) were scrutinized, encompassing articles up to 31 December 2024. Specifically, the inclusion criteria targeted studies relevant to MPs, NPs and technological innovations in drug discovery. These studies underwent careful evaluation and critical analysis and were classified with precise information. References from included articles were also screened to ensure a thorough literature review. The search utilized the following terms: “medicinal plants”, “bioactive compounds”, “natural products”, “traditional medicine”, “pharmacognosy”, “drug discovery”, “drug development”, “cutting-edge techniques”, and “omics technologies”. Unavailable full-text articles and articles not written in English were excluded.

## 3. Bridging Traditional Medicine and Modern Medicine

### 3.1. Traditional Medicine and MPs

The exploration of MPs and conventional healing methods has a long-standing history. Humans have utilized herbal medicines containing bioactive compounds at least since 2600 BC [40] and likely much earlier [41]. Also, archaeological evidence indicates the application of herbal remedies in paleolithic periods approximately 60,000 years ago to treat diseases or improve their symptoms [42].

Traditional medicine refers to a variety of health practices and approaches that utilize medicines derived from MPs and are founded on knowledge and beliefs about preventing, diagnosing and treating diseases or preserving well-being [43]. Traditional medicine is closely related to traditional knowledge of indigenous people and local societies, often lacking formal documentation and practical demonstrations [44]. A significant part of the population in developing countries uses traditional medicines, either because they are more acceptable from a cultural and spiritual standpoint or because there is limited access to modern health care services [45]. Cultural groups that have a close relationship with nature continue to explore and preserve inherited knowledge. Such knowledge, which deals with the direct interaction between children and the elderly in a community, can only be preserved orally. This fact necessitates close and prolonged family contact across generations [46]. Due to external economic and cultural forces, traditional knowledge has been replaced by industrialization and growing urbanization [47]. Furthermore, in some cases, traditional knowledge has gradually disappeared, because of cultural disruption and the expense of development.

In order to ensure the preservation of traditional knowledge, pharmacognosy, ethnobotany and ethnopharmacology are crucial disciplines that consider MPs’ scientific applicability. These three scientific disciplines are interconnected and essentially investigate the botanical origin, chemistry, biological effects and application of bioactive compounds derived from MPs [48]. It is challenging to comprehend them separately, as in many instances, studies in pharmacognosy, ethnobotany and ethnopharmacology have overlapping goals and can be beneficial methods for bioactive compound discovery [49]. The above-mentioned three disciplines may be utilized interchangeably to communicate a similar concept, although there are differences among them.

Pharmacognosy utilizes various approaches to investigate the therapeutic capability of MPs. These include the precise identification of plant species, phytochemical assessment focused on the extraction and identification of bioactive compounds, and evaluation of the potential pharmacological properties. Traditional techniques, alongside modern methods provide guidance for drug development [50]. Ethnobotany can be described as the scientific study of people’s interactions with plants. The goals of ethnobotany include enhancing knowledge of plant biodiversity [51], elucidating the cultural significance of plant sources [52], and investigating biodiversity for novel sources of NPs with pharmaceutical potential [53]. Moreover, ethnopharmacology is a multidisciplinary research field focused on the observation, description and experimental analysis of indigenous bioactive compounds and their biological effects [54]. Validating traditional preparations through pharmacological findings on native drug preparations or by isolating bioactive compounds is the goal of this field. Research into the ethnopharmacology of MPs typically includes conducting ethnobotanical surveys, which must be focused on indigenous stories and practices that serve as the basis for numerous MP-derived drugs [55].

### 3.2. NPs in Traditional Medicine

Research indicates that traditional medicines sourced from MP extracts tend to be clinically effective and cost efficient, with fewer adverse effects compared to modern pharmaceuticals [56]. Numerous examples of powerful and effective drug bioactive compounds rooted in traditional medicine have been documented by researchers in clinical laboratories (Table 1).

### 3.3. NPs in Modern Medicine

Environmental and cultural shifts, along with the transition from subsistence to market economies, have significantly impacted all facets of traditional medical practices. The excessive harvesting of MPs has caused resource degradation, reduced biodiversity, and, along with urbanization and other social processes, led to the loss of indigenous medical knowledge and practices. Additionally, the commercialization of traditional medicine to develop new pharmaceuticals for modern markets may ultimately lead to the decline of traditional medicine [119]. Although modern medicine has overshadowed the use of MP extracts, NPs are still the focus of pharmaceutical research due to the inability of other drug development approaches to provide numerous purified bioactive compounds in significant therapeutic applications [120]. Indeed, some NPs are being intensively tested for their therapeutic effects, such as artemisinin and others (Table 1).

The combination of biotechnology and synthetic biology presents challenges to researchers involved in NP drug development with improved therapeutic effects [121]. Comprehensive information on the pharmacological, metabolic and genetic properties of NPs is made possible by bioinformatic analysis, relying substantially on well-curated reference panels [122,123]. Finally, current advances in the fields of organoids and of microfluidics technology can be used to properly test new bioactive compounds on tissue and cells [124,125].

## 4. MP-Derived Metabolites

Medicinal plants contain NPs that are produced through both primary and secondary metabolic pathways (Table 2). NPs are not products of primary metabolism, a process for growth and development, but rather of chemical bioactive compounds that arise from intermediates in primary metabolic pathways [126]. SMs are located in various MP organs, with concentrations that vary based on their origin and transport mechanisms, many of which remain poorly understood across different MP species [127]. These potent bioactive compounds also serve as critical chemical signals that facilitate communication between MPs and symbiotic organisms. Secondary metabolites, which are mainly derived from primary metabolites, accumulate at different cellular, tissue and organ levels through various biosynthetic routes [16].

Recent promising efforts have been directed at enhancing the discovery of secondary metabolites and their applications by focusing primarily on the following aspects: (a) identifying possible signaling pathways that are directly or indirectly involved in the production of certain target NPs [128], (b) assessing and analyzing overall gene expression under varying conditions to investigate the gene transcripts related to NPs for a better understanding of their mechanisms of action [129] and (c) exploring genetic strategies to regulate specific transcription factors, such as modifying regulatory genes to improve the biosynthesis of targeted and desired NPs [130]. In addition to uncovering new chemical entities for therapeutic purposes, NPs serve as a vital basis for the identification of lead bioactive compounds that can be modified structurally to develop innovative and more efficient therapeutics [131]. Despite the complexity and variety of chemical structures found in MPs, secondary metabolites are believed to demonstrate superior biological compatibility and drug-like characteristics compared to compounds originating purely from synthetic processes [132]. Table 2 presents the classification of plants’ metabolites, their functions and mechanisms of action as well as some examples.

### 4.1. Chemical Diversity of Secondary Metabolites

A unique feature of plant-derived NPs is their extensive diversity of secondary metabolites, which results in remarkable chemical variability. Indeed, the diverse array of chemical entities present in SMs sourced from MPs has proven to be an invaluable resource for discovering and developing new pharmaceuticals. The abundance of secondary metabolites makes them an ideal source for identifying potential medication candidates. Recently, 8182 flavonoids followed by 160,000 alkaloids and 33,000 terpenoids have been documented in the dictionary of NPs [133]. Pharmaceutical researchers have the chance to explore this vast assortment of molecules to discover new molecules that may have therapeutic applications [134]. These molecules include a wide range of groups, with the following groups being the most important (Table 3).

### 4.2. Biosynthesis of NPs

The biosynthesis of NPs generally involves a series of enzymatic reactions that transform MPs into a variety of structurally diverse secondary metabolites. A significant obstacle in harnessing NPs lies in comprehending their biosynthetic pathways, which is essential for applying bioengineering techniques to facilitate large-scale production. Despite the extensive research, elucidating the biosynthetic pathways of any NP presents a considerable challenge due to the complex network of metabolic pathways [148].

**Figure 1:**
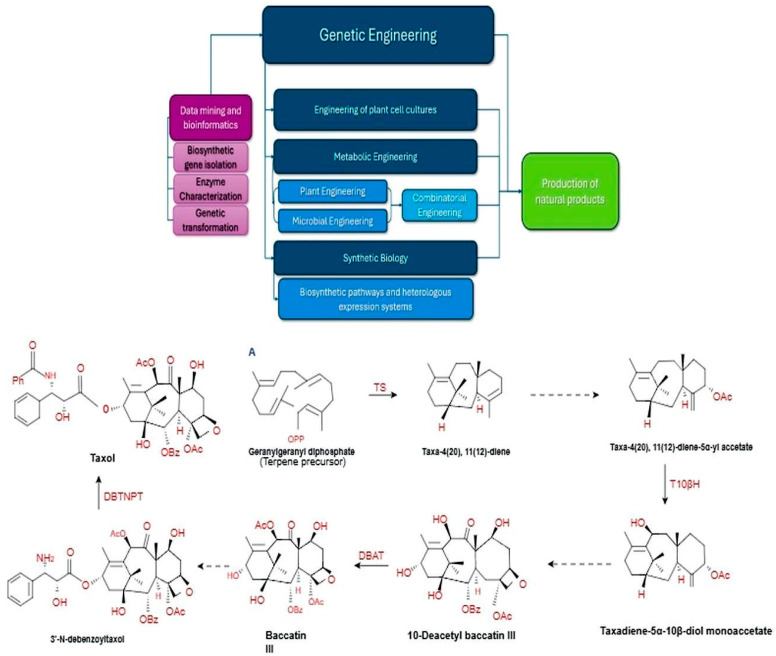
Strategies for biosynthesis of natural products based on “omics” technologies. (A) An example of the key intermediates of taxol (tetracyclic diterpenoid) biosynthesis in Taxus brevifolia, using synthetic biology [21,139]. Enzyme-coding genes are represented by the solid arrows. TS: Taxadiene synthase, T10βH: Taxoid 10β-hydroxylase, DBAT: 10-Deacetylbaccatin III-10-O-acetyltransferase, DBTNBT: 3′-*N*-debenzoyl-2′-deoxytaxol *N*-benzoyltransferase. The dashed arrows correspond to intermediates that were not included. This figure was created using Marvin (19.21.7) by Chemaxon (https://www.chemaxon.com).

The limited quantity of NPs in MPs, coupled with the rising need for these bioactive compounds, has prompted exploration into chemical and/or biotechnological synthesis. However, complete chemical synthesis of NPs has been determined to be challenging, and complex procedures are financially unfeasible for large-scale production and can contribute to environmental concerns. Additionally, isolating the desired bioactive compounds proves to be impractical and necessitates specific separation from a multitude of bioactive compounds in MPs, particularly those with similar structures and yields [149]. To address these challenges, genetic engineering, metabolic engineering, plant cell culture, and synthetic biology techniques that utilize various “omics” technologies have enabled cost-effective, sustainable, and well-regulated approaches for the production of active compounds from MPs (Figure 1).

Relying on genetic engineering strategies, a crucial first step in increasing NP production is the identification of genes, enzymes, and metabolites that are part of biosynthetic pathways through transcriptomics, genomics, proteomics and metabolomics technologies [25].

Metabolic engineering serves as a foundational technology for refining industrial fermentation processes by implementing targeted genetic modifications to produce bioactive compounds. The enhancement of NPs production is frequently achieved by reconstructing biosynthetic pathways in heterologous microorganisms to create engineered, genetically manageable microbial strains or in plants. The success of metabolic engineering relies on the improvement of strains created through genetic engineering, based on a thorough analysis of cellular functions [150].

Plant cells generate various chemicals found in the parent plant in vitro. In contrast to the labor-intensive, expensive, and environmentally harmful process of extracting NPs from MPs, plant cell culture offers a renewable, large-scale, and easily scalable source of these products. This method aids in producing bioactive compounds in a controlled environment, free from microbial and insect contamination, and is not affected by climate change or other environmental variables. Nonetheless, the slow growth of plant cells, genetic instability and low productivity remain obstacles to their use in NP production. Additionally, plant cell cultures can also serve as a platform to investigate biosynthetic pathways and the molecular mechanisms related to bioactive compounds [151].

The synthetic biology of MPs can enable the targeted and efficient heterologous production of medicinal active compounds by elucidating the biosynthetic pathways of pharmacological bioactive compounds and reconstructing these pathways and metabolic networks within heterologous expression systems. The research approaches in synthetic biology encompass the investigation of biological components involved in the metabolic pathways of pharmacological bioactive compounds, the creation and standardization of the biological components, the selection and modification of heterologous expression cells, the integration of the metabolic pathways, and the production of active compounds. The successful commercial synthesis of pharmaceuticals, such as vindoline, artemisin and paclitaxel, has demonstrated the potential of synthetic biology for large-scale production of MP-derived medicines [27,152]. Moreover, one notable instance of this approach is the successful heterologous expression of artemisinic acid in yeast, producing yields suitable for industrial production. This can then be transformed into the antimalarial drug artemisinin using a chemical source of singlet oxygen [153]. In yeast, the reconstruction of the biosynthesis of the opium alkaloid noscapine, a cough suppressant, was achieved by utilizing over 30 genes from various organisms, including plants, bacteria, mammals and fungi [154]. The integration of biosynthetic genes from various pathways within a single fungal host has also successfully led to the creation of new bioactive compounds with different or improved activities [155]. Rapid developments in plant genomics, transcriptomics and proteomics, together with the recent advent of metabolomics and new molecular biology and analytical chemistry techniques, will greatly enhance the engineering of MP biosynthesis [156]. In addition, synthetic biology and gene editing technologies [157] provide valuable, flexible and effective tools for the research, development and commercialization of NPs derived from MPs.

### 4.3. Pharmacological Properties of NPs

Research has demonstrated that NPs possess a variety of potential biological effects against numerous diseases and disorders and are notably effective [158]. It is estimated that only 15% of the approximately 250–400 thousand plant species worldwide have undergone phytochemical research, with a mere 6% systematically evaluated for biological activity [159]. Over 60% of medications used in clinical settings include natural compounds or their derivatives, with more than 120 chemical entities originating from MPs utilized to treat life-threatening conditions [160].

#### 4.3.1. Antimicrobial Properties

Some of the most prevalent NPs that exhibit antimicrobial properties against various pathogenic microorganisms include saponins, flavonoids, thiosulfinates, glucosinolates, phenolics, alkaloids and organic acids. The antimicrobial efficacy of NPs largely depends on their chemical structure, primary bioactive compounds and effective dosage. Specifically, certain terpenoids and some phenolic compounds are regarded as the main MP compounds demonstrating significant antimicrobial properties. Moreover, several essential oils containing phenolic compounds exhibit notable antimicrobial properties against a variety of pathogenic microorganisms [161]. Examples of the antibacterial, antiviral and antifungal properties of MP extracts or NPs are discussed below (Table 4).

Non-phenolic essential oils extracted from oregano, clove, rosemary, parsley and sage also display significant antibacterial effects against both Gram-positive and Gram-negative pathogenic strains [171,172]. Except for *Streptococcus pyogenes*, all other pathogens affecting the respiratory tract are sensitive to essential oils extracted from lemon grass (*Cymbopogon citratus*), lemon balm (*Melissa officinalis*), cinnamon bark (*Cinnamomum verum*), and thyme (*Thymus vulgaris*) [173]. Recently, specific MP extracts and NPs, including alkaloids, polysaccharides, and flavonoids, have demonstrated effective results against *Helicobacter pylori* infections. *Daucus carota* (carrot) seed oil has shown the highest effectiveness against *Helicobacter pylory* in vitro [174].

Recently, various studies have been conducted to identify new NPs that can act as antiviral agents [175]. Indeed, numerous NPs have demonstrated in vitro antiviral properties, although their efficacy in vivo is often lower [176]. Two MP-derived antiviral compounds, (+)-Calanolide A and SP-303, are currently in clinical development. (+)-Calanolide A, extracted from the Malaysian rainforest tree *Calophyllumlangigerum*, has shown significant HIV reverse transcriptase inhibitory activity [177]. SP-303, sourced from the latex of the Latin American plant *Croton lechleri*, has demonstrated potential in vitro activity against Herpes simplex viruses (HSV) [177]. Various alkaloids, including Citrusinine I (derived from *Citrus* sp.), Atropine (from *Atropa belladona*), and Scopolamine (from *Datura stramonium*), have shown antiviral properties against numerous viruses, such as HSV [178]. Moreover, research has suggested that certain essential oils possess activity against the COVID-19 virus due to their various properties, such as antiviral, anti-inflammatory and immunomodulatory effects [179]. Much of the research on antiviral NPs has focused on inhibiting different enzymes associated with the viral cycle.

Natural products exhibit antifungal properties, as they can induce cytotoxic effects in fungi by impairing cell membrane permeability and inhibiting enzymes involved in their growth. Various MP extracts have demonstrated antifungal and anti-mycotoxigenic properties, along with antioxidant effects against harmful fungal strains, such as *Fusarium vericillioides*, *Aspergillus ochraceus*, and *Aspergillus flavus* [180]. Extracts from wild stevia exhibit significant antifungal, anti-mycotoxigenic and antioxidant properties against species such as *Aspergillus flavus*, *Aspergillus ochreaceus*, *Aspergillus niger*, and *Fusarium moniliforme* [181]. Additionally, EOs possess the capability to inhibit the growth of mycotoxigenic fungi, including *Aspergillus flavus* and *Fusarium moniliforme.*

#### 4.3.2. Antioxidant Properties

Antioxidants are substances that can prevent oxidation, a chemical process that generates free radicals and chain reactions, potentially harming cells. Antioxidants are beneficial for enhancing the quality of processed foods and preventing spoilage [182]. NPs that display potential antioxidant properties can serve as natural alternatives to numerous synthetic substances for improving food quality and extending shelf life [183,184]. Several NPs, such as flavonoids, lignans, vitamins, carotenoids and terpenoids, are notable for their strong antioxidant activities [185]. Aqueous tea extract is among the most widely used natural antioxidant in the food industry due to its high content of catechins, tannins, and flavonoids, which do not alter the flavor of food [186]. Additionally, broccoli, cabbage, tomatoes and lettuce produce several potential antioxidants, which assist in the solubility of lipid compounds and mitigate cell damage caused by significant oxidative stress [187]. Also, various EOs derived from oregano, marjoram, thyme, verbena, sage and rosemary are recognized as abundant sources of natural antioxidants [188].

#### 4.3.3. Anti-Inflammatory Properties

Inflammation is a complicated biological protective response of bodily tissues to microbial infection, tissue damage, or irritants, involving immune cells, blood vessels and molecular mediators [189]. Various studies have documented the anti-inflammatory properties of certain MPs, such as *Curcuma longa*, *Zingiber officinale*, *Rosmarinus officinalis* and *Borago officinalis*, which have promising clinical applications [190]. Recently developed NPs as anti-inflammatory medicines offer a rich resource ranging from comprehensive explanations to molecular docking methods for naturally occurring compounds with anti-inflammatory effects [191]. The development of anti-inflammatory substances based on NPs, including polyphenols, terpenes, fatty acids, and numerous other bioactive compounds, has shown remarkable effectiveness. Specifically, it is reported that many NPs, such as moupinamide, capsaicin and hypaphorine, derived from *Zanthoxylum beecheyanum*, chili pepper and *Erythrina velutina*, respectively, may serve as new anti-inflammatory medicines [192].

#### 4.3.4. Anticancer Properties

Numerous NPs have been identified as having potential anticancer properties because of their ability to mitigate oxidative stress and inflammation that can harm DNA, subsequently leading to cancer development [193]. NPs, including irinotecan, vincristine, vinblastine, etoposide, and paclitaxel, were sourced from plants [194]. Additionally, capsaicinn, found in Capsicum has shown potential as an anticancer, tumor-suppressing, chemopreventive and radiation-sensitizing agent across various cancer models [195]. Due to its ability to inhibit carcinogenic activity and induce apoptosis in multiple cancer cell lines in vitro and in rodent studies, capsaicin is explored as a cancer treatment [196]. Catechins are naturally occurring dietary polyphenols. The primary components of green tea include Catechin (C), epicatechin (EC), epigallocathechin (EGC), and epigallocatechin-3-gallate (EGCG). EGCG can augment the effects of several anticancer medicines [197]. Lycopene functions as an antioxidant, offering protective benefits against a range of diseases, such as cancer, hypertension, osteoporosis, cardiovascular conditions, and neurodegenerative disorders [198]. Isoflavones derived from soy, specifically genistein, have been recognized for their significant anticancer effects against conditions such as lymphoma, leukemia, and breast, prostate, gastric and non-small cell lung cancer [199]. Various MPs, including ginger, capsicum, curcumin, clove, rosemary, sage, oregano and cinnamon, are abundant in antioxidants due to a high concentration of phenolic compounds, and have been demonstrated to counteract damage caused by reactive oxygen species in multiple human cancers [191].

### 4.4. Environmental Factors in the Formation of SMs

Medicinal plants play a vital role in numerous aspects of human life, but their growth and development can be influenced by environmental factors. Each plant species requires specific environmental conditions for optimal growth, making these factors significant [200]. Environmental stressors not only lead to structural and anatomical changes in MPs but also can negatively influence the production, growth and overall productivity of MPs. Understanding the complex relationship between environmental signals and the production of SMs is crucial for enhancing the cultivation conditions of MPs and for the sustainable production of bioactive compounds for pharmaceutical uses. Furthermore, clarifying the molecular mechanisms driving these responses can help devise strategies for manipulating the biosynthesis of SMs to achieve preferred traits in crops.

Climate change, along with temperature fluctuations, drought conditions, and increasing levels of CO_2_ in the atmosphere, is anticipated to impact the synthesis of secondary metabolites in plants. These environmental factors are significant contributors to changes in MPs regarding the production and release of flavonoids, phenolic acids, and volatile bioactive compounds [201]. However, the implications of these metabolic changes on plant survival and adaptability are still lacking, which raises potential concerns regarding challenges related to the industrial extraction and purification of medicinal products. Furthermore, climate change could escalate into a more severe issue for the MP community, presenting potential challenges for users, harvesters, and manufacturers of MP species, as well as affecting their availability as raw materials [202]. Furthermore, the impacts of changes in land use are causing biodiversity loss, which endangers the long-term sustainability of ecosystem functions on a global scale [203]. Additionally, climate change has already elevated the risk of species extinction, and its consequences further exacerbate the impacts of land-use changes, with forecasts indicating negative effects on biodiversity in the coming decades [204].

#### 4.4.1. Biotic Factors

Endophytes—microorganisms capable of invading healthy plants without causing any illness—may trigger or stop the production of secondary metabolites, suggesting that using endophytes as a form of green fertilizer could be an effective and sustainable method to enhance the production of secondary metabolites [205].

Some secondary metabolites, identified as phytoalexins, exhibit antimicrobial properties. When MPs promote their defense mechanisms against pathogens, various metabolic pathways are activated, and the high demand for secondary metabolites triggers their rapid production [206].

To defend themselves against herbivores, MPs produce secondary metabolites. These secondary metabolites regulate the signaling pathways that are involved in plant defense and facilitate defensive roles. Herbivores trigger a complex cascade of events that culminates in the synthesis and accumulation of secondary metabolites [207].

#### 4.4.2. Abiotic Factors

The physical characteristics of light affect the synthesis of SMs in various MP species (Table 5).

Moreover, secondary metabolite production is augmented by ultraviolet light exposure. A rise in ultraviolet-B radiation generates a greater amount of essential oils and phenolic compounds. Additionally, light regulates both the volume and quality of secondary metabolites produced by MPs. For example, increased sunlight exposure encourages the accumulation of high levels of coumarin in *M. glomerata*. Similarly, light intensity significantly impacts the process of terpenoid production and the expression of genes that code for terpenoid synthase [216].

Temperature is a critical environmental factor that influences enzyme activity and metabolic pathways, thus affecting the production of secondary metabolites [217]. Studies have shown that fluctuations in temperature can alter the diversity, concentration, and composition of secondary metabolites generated by marine organisms, microorganisms, and MPs. An increase in temperature accelerates the aging of leaves in *Panax quinquefolius* and promotes the accumulation of secondary metabolites in its roots. It is important to note that although thermal stress can stimulate or inhibit the production of secondary metabolites in MPs, it significantly retards plant growth and leads to senescence.

Carbon dioxide is recognized as adversely affecting the physiology of MPs. MPs adapt to environmental changes through metabolic flexibility, but this adaptability also influences the secondary metabolites that underlie their medicinal properties. For instance, *Hypericum perforatum* L. (St. John’s Wort) was subjected to elevated CO_2_ levels and demonstrated growth enhancements after 140 days compared to normal conditions [218]. 

Drought represents a significant environmental stressor capable of modifying the physiological and biochemical attributes of MPs while raising the levels of secondary metabolites. The capacity of different MP species to endure drought shows considerable variability. Drought conditions are defined by low available water levels combined with high temperatures [219]. In *Glechoma longituba*, insufficient water leads to a decrease in total flavonoid content, with a field capacity water treatment of 80 to 85% being optimal for maximizing total flavonoid levels [220].

The presence of salt in the environment promotes the production of various SMs, including phenols, terpenes and alkaloids. Certain MP species exhibit increased concentrations of anthocyanins in response to salt stress. A correlation is observed between higher salinity and increased polyphenol content in specific plant tissues. In Chamomilla’s (*Matricaria chamomilla*), essential oil features are adversely impacted by salinity [221]. Salt stress leads to an elevation in the levels of the alkaloids reserpine and vincristine, which are recognized as MP stress secondary metabolites in *Rauvolfiatetraphylla* and *C. roseus*, respectively.

Soil fertility is influenced by several elements, particularly nitrogen, which affects primary metabolism and the production of secondary metabolites. This significantly impacts the quality of the raw materials produced by MPs as well as their overall growth and development. A notable inverse relationship was found between the levels of nitrogen and phosphate and the amount of flavonol in the seeding tissues of Arabidopsis MP. The ability of *Labisia pumila Benth*’s to produce total phenolics and flavonoids was greatly influenced by the nitrogen levels in its environment. Moreover, increased fertilization was linked to a slight enhancement in antioxidant activities [222].

## 5. NP Drug Discovery and Development

The ultimate goal of all drug discovery efforts is to identify the most promising NPs that can serve as therapeutic agents for treating medical conditions [223]. In the initial phases of the drug design process, researchers must isolate and purify compounds from their natural origins using various methods tailored to the structural diversity, stability and required quantity of the NPs. High-throughput screening techniques have been employed to evaluate the NPs against specific targets. After conducting essential biochemical and pharmacological assessments, the promising NPs for these specific targets are chosen. During this stage of drug design, many NPs may lack sufficient selectivity for their intended target molecule. To enhance selectivity, scientists adjust the structures of the NPs according to anticipated structure–activity relationships. If these modifications successfully improve selectivity, the promising bioactive compounds progress to in vitro and in vivo testing in appropriate disease models [224]. If a compound successfully passes preclinical evaluations, it moves on to human clinical trials. Clinical trials evaluate the safety and effectiveness of the compound in patients, generating the necessary data for regulatory approval and market clearance. Once clinical trials and regulatory approval are successfully completed, the compound is released as a new drug for medical use [223].

### 5.1. Discovering NPs from MPs

**Figure 2:**
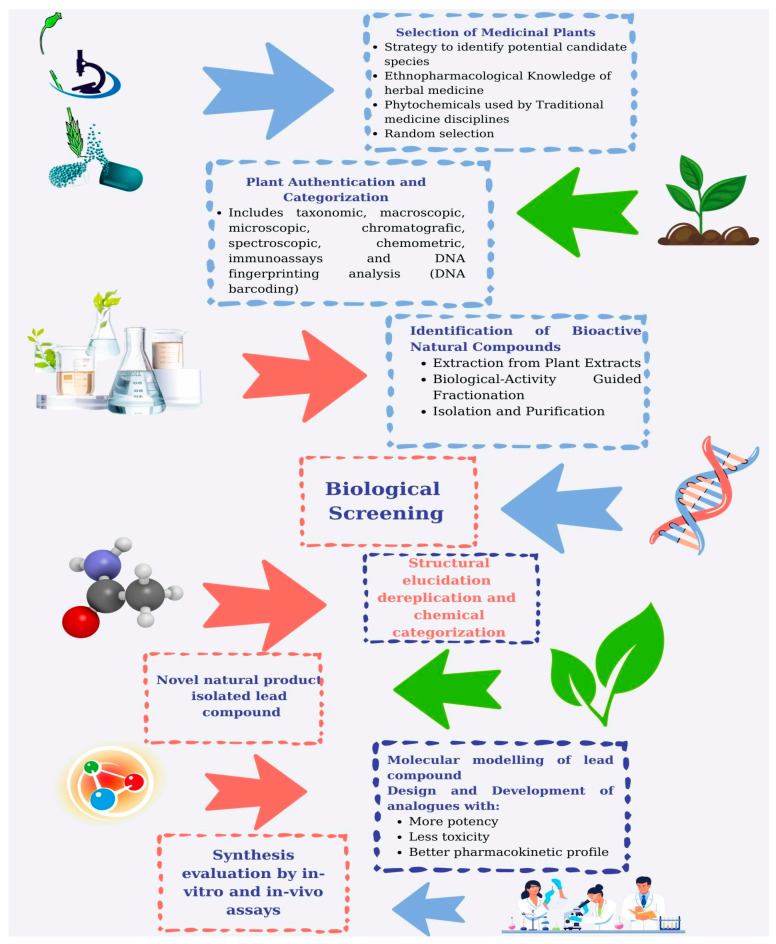
Steps related to the natural product discovery and drug development process from medicinal plants. This figure was created using canva.com.

The journey of discovering and developing therapeutics sourced from MPs is a complex and varied process that involves several critical stages, each playing a key role in converting the potential of MP-derived compounds into effective medicines (Figure 2).

#### 5.1.1. Plant Selection

In the initial phases of drug development, the expertise of botanical specialists and ethnobotanists is vital for identifying and gathering plant species that may have therapeutic properties. The selection of MPs can be influenced by various information sources, including traditional knowledge often derived from indigenous and local communities, or can be performed randomly. Ethnobotanical studies and field surveys may be conducted to spot potential plant candidates with promising attributes. This collection process serves as a crucial first step toward accessing the diverse array of bioactive compounds present in the plant kingdom [225].

#### 5.1.2. MP Authentication

The quality and authentication of harvested raw materials are the fundamental starting point in the creation of NPs. MP material authentication can be accomplished using one or more methodologies, including taxonomic, macroscopic, chromatographic, spectroscopic, chemometric, immunoassays, and DNA barcoding fingerprinting techniques [226]. The quality may be impacted by several factors, including variations between species or within a species, environmental conditions, seasonal changes, harvesting time and methods, geographic origin of the herb, and the plant part utilized, as well as storage and processing techniques [227].

#### 5.1.3. Extraction

Once the plant material is collected, the next step involves extracting bioactive compounds from the MP matrix. Different methodologies can be employed for this purpose, depending on the chemical makeup of the compounds and the nature of the plant material [228]. Common extraction methods include maceration, where the MP substance is soaked in an appropriate solvent, Soxhlet extraction, which facilitates continuous solvent extraction, and decoction, which is an extraction technique for heat-stable bioactive compounds [229]. Eco-extraction methods, or green extraction techniques, focus on minimizing environmental impact and enhancing sustainability when extracting bioactive components from MPs [230]. Table 6 presents the common and green extraction methods used for extracting bioactive substances from MPs.

Current trends in green extraction methodologies indicate that integrating multiple extraction techniques into a single procedure can lead to better results. Indeed, the application of innovative and combined technologies improves extractability, leading to higher extraction rates, reduced impurities in the final extract, preservation of the thermal-sensitive compounds, the use of various inorganic solvents, and overall lower energy consumption [253].

#### 5.1.4. Isolation and Purification

The material extracted from MPs typically contains a mix of various compounds, including both bioactive and non-bioactive substances. To isolate the specific active compounds, the mixture is subjected to fractionation and purification processes. Recently, methods linked to biological activity-guided fractionation, along with chromatographic separation techniques, have been widely utilized for these processes. The biological activity-guided fractionation relies on the biological activity of the fractions rather than targeting a specific class of compounds, involving a systematic separation of the MP extract. Based on physicochemical characteristics and biological activity screening, subsequent fractionation and evaluation take place [254]. Chromatographic techniques often involve column chromatography, which separates bioactive compounds based on their chemical properties, and HPLC, which provides a high-resolution method for sorting and quantifying molecules. Other methods, such as solvent partitioning and crystallization, may also be utilized to refine the desired bioactive compounds [255].

#### 5.1.5. Bioassays

Bioassays serve as the main tool for understanding bioactivities derived from natural sources. They are most commonly utilized in the initial stages of bioactive compound discovery to assist in the isolation/purification of fractionation processes to isolate and identify pure bioactive compounds, requiring them to be designed for high-throughput capacity, rapid execution, ease of performing, and cost effectiveness [256,257]. Once bioactive compounds have been purified and identified, the subsequent critical phases involve evaluating their pharmacological effectiveness and safety. In vitro and in vivo bioassays can also be employed to determine the efficacy, safety and possible mechanisms of action of the substances. This generally necessitates a collection of bioassays that are highly specific and precise, and tend to be time-consuming and costly [256]. In vitro tests are conducted in controlled laboratory settings, commonly utilizing cell cultures or isolated biochemical systems, to examine the effects of the compounds at the cellular and molecular levels [257]. Conversely, in vivo preclinical studies assess the substances in live organisms to provide insights into their physiological and toxicological properties. In the case of clinical trials, four phases must be completed prior to obtaining drug approval. 

### 5.2. Innovative Technological Approaches in NP Drug Discovery and Development

To effectively develop new medication, it is essential to implement innovative and multidisciplinary approaches that support NPs for drug discovery and development in clinics. The integration of these approaches is likely to produce new medications capable of addressing current health issues [258]. Notably, systems biology encompasses a range of tools that are frequently employed in tandem. A notable systems biology workflow was described to predict how the natural sesquiterpene alkaloid, huperzine A, might influence molecular elements associated with Alzheimer’s disease. The process started by identifying the most likely macromolecular targets of huperzine A. The results were then correlated with proteins linked to Alzheimer’s disease using the DisGeNET gene-disease association type ontology. The selected proteins were subsequently enriched using STRING biological database to reveal affected pathways and cellular components. Further insights into the molecular interactions between huperzine A and acetylcholinesterase, a crucial enzymatic target in Alzheimer’s disease, were obtained through protein–ligand docking simulations. The primary outcome of this computational analysis suggested that huperzine A likely influences Alzheimer’s disease through various synaptic neurotransmitter and metabolic pathways, rather than through a direct effect on acetylcholinesterase [259].

The potential for new drug discoveries depends on various innovative technologies, such as high-throughput screening, which enable the testing of potentially bioactive compounds on a large scale and speed up the identification of molecules for subsequent development. High-throughput screening is an effective screening approach that utilizes an automated operating system for conducting experiments and collects data using rapid and sensitive detection instruments at both the molecular and cellular levels. The technical system operates with the support of relevant databases, allowing for the concurrent testing of millions of samples, after which the experimental data is analyzed and processed via computers [260]. An example of this target screening approach includes identifying glucagon-like peptide-1 receptor activity by examining the Astragalus propinquus Schischkin and Panax notoginseng compound preparation [261].

Another target-screening approach is the clustered regularly interspaced short palindromic repeats (CRISPR) along with CRISPR-associated protein 9 (CRISPR-Cas9 system) which has been employed to modulate the genes that govern critical biosynthetic pathways in vitro systems of plants to improve secondary metabolites [262] It has practical applications in modifying desirable bioactive compounds that are important for therapeutic purposes in MPs. For example, the CRISPR-Cas9 gene editing tool was utilized to disrupt the SmCPS1 gene in *Salvia miltiorrhiza*, a traditional Chinese medicinal herb commonly used for treating cardiovascular and cerebrovascular conditions [263].

Systems of wide approaches, such as network pharmacology, computer-aided NP drug discovery (virtual screening, molecular docking, molecular dynamics simulation and artificial intelligence), pathway analysis, molecular networking and nanobiotechnology, can be employed in discovering drugs from natural sources. Table 7 presents the most innovative methods for NP discovery and development.

#### 5.2.1. Network Pharmacology

The network pharmacology approach offers a new strategy for drug discovery, especially for historically significant products utilized in Traditional Chinese Medicine or Ayurveda [308]. In contrast to conventional approaches that focus on single-target drugs, network pharmacology consolidates extensive data obtained from diverse sources and provides a comprehensive view of interactions between secondary metabolites and disease, which frequently engage multiple signaling pathways and biological targets [309]. The holistic and integrative nature of data gathered through network pharmacology and related techniques, such as pathway analysis and molecular networking, presents a unique analytical framework in the realms of biology and pharmacology, facilitating an enhanced comprehension of molecular mechanisms and the identification of potential therapeutic strategies. Network pharmacology can also be utilized to identify the class of active secondary metabolites present in plant materials prior to optimizing the extraction method, or to pinpoint those that contribute to the biological activity of the entire extract. For example, the combination of in vivo assays with UPLC-qToF/MS analysis, along with a network pharmacology approach, resulted in the identification of six bioactive compounds (aloesin, aloenin, aloin B, aloin A and aloe-emodin) [310].

#### 5.2.2. Computer-Aided MP Drug Discovery

Computational techniques have become a valuable tool for discovering and optimizing NP therapeutics. Specifically, in silico screening approaches assist in enhancing bioavailability, predicting drug efficacy and identifying potential side effects [311]. By utilizing computational techniques, researchers can effectively identify bioactive compounds that are bioactive against drug-resistant targets. For instance, allicin, derived from garlic, targets bacterial efflux pumps and inhibits biofilm formation [312].

##### Virtual Screening

Within the framework of network pharmacology, one approach that allows for the hierarchical filtering of drug candidates based on their capacity to influence multiple biological targets within a network is virtual screening. Virtual screening employs a combination of computational techniques, and can be applied in various manners, either searching for bioactive compounds that exhibit structural similarities to known references or evaluating both the three-dimensional structure of the ligand and the binding affinity of new molecules for biological targets related to disease [313]. The latter approach represents structure-based virtual screening, which encompasses techniques such as structure-based pharmacophore prediction, molecular docking and molecular dynamics simulations [314].

##### Molecular Docking

Molecular docking is among the most commonly utilized techniques in computational drug discovery. It investigates the behavior of molecules at the binding site of a target protein. Molecular docking is frequently employed to model the interactions between natural bioactive compounds and various enzymes. The goal is to discover and design effective and selective enzyme inhibitors by screening molecules found in MP extracts and to elucidate the potential mechanisms underlying the observed biological activity. Typically, the most prevalent molecules in the extract, or isolated bioactive compounds, are docked into proteins, whose inhibition may enhance bioactivity. For example, to elucidate the antimicrobial activity, key biocompounds of the *Amaranthus lividus* extract, such as gallic acid and phytol, were screened for interactions with aquaporin and arginase. Aquaporins, which function as pumps in the cell membrane, are targeted by numerous bactericidal agents, while arginase is an enzyme that plays a role in the metabolism of arginine, a critical pathway in bacterial pathogenesis. Both bioactive compounds were predicted to form hydrogen bonds and hydrophobic interactions with amino acid residues of the target macromolecules, but with different binding energies and inhibition constants [315].

##### Molecular Dynamics Simulation

Molecular dynamics simulation may also significantly contribute to refining docking or virtual screening results, as biomolecules within the human body are dynamic rather than static conformations used in traditional structure-based drug design techniques. Molecular dynamics simulation predicts the molecular and structural alterations of biomolecules due to inter- and intramolecular forces, making it vital for drug discovery research [316]. Numerous software tools are available for molecular dynamics simulation due to their powerful computational algorithms [317]. Additionally, molecular dynamics simulations can enhance structural models by incorporating dynamics and atomic-level movements [318]. The application of these simulations is crucial for predicting the binding affinity of flavonoids to G-quadruplex DNA, which plays a significant role in cancer treatment [319].

##### Artificial Intelligence and Machine Learning

The algorithms of artificial intelligence and machine learning possess significant potential to enhance our comprehension of phytochemistry and its medical applications. Researchers have contributed valuable perspectives to network-based approaches [320]. These advanced computational tools enable researchers to analyze the complex relationships between the molecular characteristics of NPs and their biological properties, thereby pinpointing potential therapeutic targets. Furthermore, machine learning paves the way for the creation of therapies tailored to specific NPs, predicting possible reactions unique to individual patients [321]. In one study, two machine learning models showed that demethylzeeylasteral, derived from the *Tripterygium wilfordii* Radix, demonstrated potent anti-obesity effects in vitro [322]. Another investigation explored a novel computational screening approach that categorized bioactive compounds by utilizing algorithms. This screening resulted in the identification of antimicrobial properties of various essential oils from *Cinnamomum sieboldii* and *Lindera triloba* against *S. aureus* [323].

#### 5.2.3. Pathway Analysis

In the field of system-wide techniques, pathways are associated with particular biological functions. Collections of genes, proteins and metabolites constitute molecular entities involved in metabolic pathways [324]. Analyzing these collections in relation to phenotypes yields crucial insights into the molecular processes contributing to the pathophysiology of diseases [325]. Pathway analysis is commonly one of the techniques utilized in developing intricate networks that illustrate relationships, such as gene–disease, drug–target, and gene–drug–target, a prime example being network pharmacology [326]. Pathway analysis was initially created to interpret transcriptomics (gene expression data from micro assay and high-throughput sequencing research) data, but has also been effectively applied to metabolomics, which are utilized to investigate metabolic pathways [325]. For example, GC-MS metabolomics-based analyses of secondary metabolite sets and pathway enrichment demonstrated that the Qianggan formula, which is utilized for managing chronic liver conditions and preventing increases in blood glucose, reduces hyperglycemia by influencing glucometabolic pathways [327].

#### 5.2.4. Molecular Networking

Molecular networking is based on the idea that a distinct structural connection exists between two molecules, which are depicted as nodes in the network. The edges of the network indicate either direct or indirect interactions among genes, proteins or secondary metabolites. Metabolite-based molecular networking offers numerous interesting applications, especially in the field of drug discovery from natural sources. It can help to identify bioactive compounds within complex mixtures or establish molecular disease signatures [328]. Furthermore, an innovative approach that combines liquid chromatography high-resolution mass spectrometry with molecular networking to evaluate the biological activity of unidentified alkaloids sourced from the relatively obscure Iceland poppy (*Papaver nudicaule*) was presented [329]. Another possible use of molecular networking is to identify bioactive compounds directly from fractioned extracts. The biological activity-guided annotation via molecular networking has emerged as a popular technique for identifying plant-derived bioactive compounds in traditional natural remedies. For instance, evodiamine, dehydroevodiamine, and schinifoline alkaloids were recognized as key markers linked to the antiproliferative effects of *Tetradium ruticarpum* fruit against HL-60, T24, and LX-2 human cell lines [330].

#### 5.2.5. NP-Based Nanobiotechnology

Nanobiotechnology allows for the direct administration of medication to targeted areas, enhancing their effectiveness and minimizing adverse effects associated with systemic drug absorption [331]. Moreover, nanobiotechnology can enhance the effectiveness of NPs by improving bioavailability. One characteristic that can render NPs less suitable for drug delivery is their lipophilicity, as they tend not to dissolve effectively in the bloodstream. The water solubility and effectiveness of such lipophilic NPs can be enhanced through nano-encapsulation. The bioavailability of highly lipophilic natural substances, such as curcumin, resveratrol and epigallocatechin gallate, can be improved via nano-encapsulation [332]. Computational techniques, known as nanoinformatics, are utilized for managing raw data, analyzing data obtained from biomedical applications, and simulating interactions between nanoparticles and biological systems [333].

## 6. Regulatory Frameworks for NPs

The primary obstacles to the development and promotion of NPs include chemo-profiling, safety assessments, quality assurance, and establishing effective regulatory frameworks. A novel regulatory framework might be beneficial to facilitate streamlined pharmaceutical development of innovative NPs, ultimately increasing the availability of safe and effective options in the market. Every nation that oversees MP medicines should implement quality assurance and control measures, such as national quality specifications and standards for MP products, good agricultural practices (GAPs), good manufacturing practices (GMPs) for MP drugs, proper labeling, manufacturing licenses, imports and wholesale activities. In a more favorable regulatory setting, a quicker process could be applied to the clinical development of these medicines, thereby enhancing the therapeutic index of traditionally utilized products [334]. The fact remains that, unlike the popular intuitive belief, the meaning of “safety” does not equate to the meaning of “natural”. Concerns regarding adverse reactions are becoming increasingly prevalent, and the assumption that MP medicines are “safe” simply due to their “natural” origins warrants clarification for the public [335]. Different regulatory authorities have varied in their requirements for toxicological data, as well as their application of clinical trial information, adverse event reports, and the historical usage of MPs as medicines in their evaluations. Another significant challenge in many countries is that regulatory information regarding MP medicines is often not shared among regulatory authorities and safety monitoring or pharmacovigilance centers [336]. Furthermore, this regulation is often not observed in many regions around the globe, particularly in developing countries, where many NPs remain unregistered and/or inadequately regulated, being sold openly in the market with minimal oversight [336].

The safety of traditional MP medicines has become a significant concern for both national health authorities and the general public. Manufacturers must ensure that their NPs are safe and properly labeled by following the policies below. To guarantee NPs’ consistency and the safety of consumers, manufacturers of MP medicine are required to follow quality control and GMPs. These practices encompass all facets of production, from sourcing raw materials to the final packaging. For example, Traditional Medicinals, a prominent MP tea brand, takes pride in its comprehensive quality control practices that exceed industry norms. GAP for MPs are also established to regulate production, ensure quality and aid in the standardization of MP medicines [337]. GAP is a comprehensive approach that uses high-quality, safe and uncontaminated raw MP products to address various challenges [338]. GAP encompasses numerous aspects, including environmental ecology, production sites, cultivation methods, collection practices and quality control [339]. In addition to regulatory adherence, there is an increasing consumer preference for MP medicines that are sustainably sourced and ethically produced. Although the regulatory landscape for MP medicines can pose challenges, it simultaneously provides opportunities for businesses to stand out through quality, transparency and ethical practices.

## 7. Problems in NP-Based Drug Discovery

The most common problem in assessing the pharmacological activity of NPs is that extracts from individual MPs contain mixtures of several bioactive compounds, which may differ in concentration or composition due to environmental variations [340]. This complicates pharmacological investigations of such mixtures since identifying which specific compound is bioactive is difficult and often necessitates adequate methods [341].

A lack of positive results in a screening assay does not invariably indicate that bioactive compounds are absent. They may be present in insufficient quantities in the crude extracts to demonstrate activity at the tested levels. Moreover, if the bioactive compound is found in higher concentrations, there might be other bioactive compounds that have opposing effects or diminish the positive effects of the active compound during the assay [342].

In vitro screening techniques encounter difficulties because some bioactive compounds that show significant activity in vitro assays may be metabolized in vivo into inactive secondary metabolites. Conversely, some extracts may only show activity in vivo due to the conversion of inactive compounds into active forms. Additionally, certain bioactive compounds can exhibit synergistic effects when they are combined with other bioactive compounds from the extract. Therefore, fractionating extracts during the purification process may result in a reduction or complete loss of bioactivity across all fractions. Despite these challenges, accurately evaluating the pharmacological properties of natural products remains essential—though it is often a complex and demanding task [340].

All contemporary methods presented in Table 7 can help in various ways to address these problems. For instance, metabolomics and proteomics may resolve the issue of activation and inactivation of biomolecules.

## 8. Challenges Associated with NP Drug Discovery

Drug discovery from NPs presents several challenges, including limited availability of bioactive compounds, difficulties in isolating and purifying them, and potential inconsistencies in quality and potency Nowadays, the process of bioassay-guided fractionation has significantly improved due to advancements in precise instruments, such as GC-MS, NMR, and HPLC-SPENMR, especially for bioactive compounds that exist in very limited amounts in the MPs [342,343].

A major challenge in MP drug discovery is the decline in plant species due to irresponsible resource utilization. For instance, the over-harvesting of plant species may result in the depletion of natural resources. This emphasizes the need for sustainable and responsible sourcing practices and preservation of MP species to safeguard both the ecosystem and the indigenous communities relying on these resources [344]. Bioprospecting methods coupled with GIS systems (Table 7) can significantly help in this direction.

Factors such as the rising use of NPs, dependence on raw material sourcing from underdeveloped regions, insufficient regulation regarding MP remedies in many countries, and growing safety concerns have heightened the awareness of the need to monitor quality and safety, as well as to improve understanding of the potential risks and benefits associated with NP medical use [345]. Clinical research can only begin once essential preclinical data on the intervention have been collected and suitable approvals and secured [346]. A notable challenge in randomized clinical trials of NP medicines is the choice of controls, which should ideally match the NP intervention class as closely as possible. The various hurdles and laboratory requirements identified for clinical trials are important considerations for numerous sectors before proceeding with a clinical investigation of their products.

A considerable number of NPs have not undergone comprehensive testing, and their applications are often insufficiently monitored or completely unmonitored. This leads to a restricted understanding of their mechanisms of action, adverse effects, contraindications, and interactions with other pharmaceutical products [336]. Recent advancements in chemical synthesis and biosynthetic engineering (Table 7) are considerably improving NP drug discovery and development, facilitating the optimization of complex properties that were previously thought to be unattainable [14]. An internationally acknowledged regulatory system, covering every aspect of MPs and NPs, from harvesting to the final product, is more than necessary to address all the aforementioned discrepancies. Such a regulatory system will not only protect the MPs but also enhance communication in the scientific community.

## 9. Prospects for NPs in Drug Discovery

Prospects for discovering drugs from NPs involve crucial issues, such as biological diversity and contemporary genomics [347]. To address the demand for new drugs and tackle clinical conditions that conventional treatments cannot resolve, biotechnology and pharmaceutical companies need to innovate new technologies. Currently, drug discovery processes that originate from natural resources mainly concentrate on the screening, isolation, purification and identification of new drug candidates. Advancing any promising lead bioactive compounds necessitates large-scale extraction or biotechnological production to make these bioactive compounds clinically applicable. Strategies to improve the selectivity and yield of extraction, including modeling solvent–bioactive compounds interactions and affinities, along with the optimization of physical parameters for non-conventional methods, should be further investigated to reduce costs and support further improvement. Furthermore, studies in drug stability, as well as research on efficiency, pharmacokinetics, and metabolic engineering, are essential to identify bioactive compounds of interest and progress them into clinically relevant therapies. Understanding the mechanisms of action of bioactive compounds is often better achieved through the in vivo screening methods. Therefore, the discovery of these compounds is limited when only in vitro assays are employed [348]. Creating in vivo effective assays, particularly for antibiotic testing, could enhance the screening of such bioactive compounds by utilizing innovative mechanisms.

MP genomics has provided a wealth of genetic resources for the research of NPs biosynthesis [349]. Advancements in genetic engineering of metabolic pathways within microorganisms and crop plants through synthetic biology are paving new avenues for leveraging plant chemistry while minimizing the risk of depleting fragile natural resources. Combining biosynthesis with both natural and enhanced catalysts taken from various MP sources will further produce a broader variety of natural-like unnatural compounds, featuring expanded pharmacological potentials [350]. Recently, DNA synthesis has allowed for the large-scale production of enzymes, which aid in constructing and modifying the biosynthetic pathways of natural products [351]. Particularly, machine learning has emerged as an effective method for clarifying the complex molecular mechanisms of intricate biosynthetic pathways. The application of these cutting-edge technologies supports the sustainable production of NPs, thereby supplying abundant chemical resources for pharmaceutical research. “Omics”-based methodologies aid in the identification of genes associated with complex NP biosynthetic pathways. Coupled with the ongoing enhancement of advanced synthetic biology tools and detailed genome-scale models of metabolism that permit superior metabolic engineering, enhancing NP-based drug development, we can now reasonably anticipate that this rapidly evolving field will provide effective solutions for marketed NPs [352].

The process of MP drug manufacturing is ongoing to implement GAP and GMPs and guarantee the standard and quality of NPs. To prevent delays in the registration of new drugs, the proper application of drug control authority regulations through harmonization is necessary. MP companies, manufacturers and entrepreneurs must know government strategies for business and market growth and actively promote high-quality, safe MP medicines that are regularly updated.

While NP-based drug discovery presents a unique opportunity for various forms of science-industry collaboration, a major challenge is that scientific and technological expertise is frequently dispersed across numerous academic institutions and businesses. Concentrated efforts are necessary to support translational NP research, which has become increasingly difficult in recent years due to a reduction in the number of large companies actively involved in NP drug discovery.

## 10. Conclusions

Natural products are diverse bioactive compounds found in MPs, and they have historically been and will continue to play a vital role as sources of therapeutic agents and as frameworks for the design and synthesis of various drugs aimed at treating diseases. Additionally, NPs contribute to the survival of MPs, while their production is influenced by environmental factors and changes in climatic conditions. Bridging traditional knowledge with modern scientific approaches is crucial for optimizing the potential of NPs in drug discovery. It is essential that the traditional applications of MPs are systematically studied and standardized to guarantee quality, safety and efficacy in NP medicines. It is also crucial to employ an interdisciplinary strategy that incorporates traditional and ethnopharmacological knowledge, ethnobotany, analytical chemistry, appropriate biological screening techniques, and modern drug development tools for successful outcomes in this field. The inclusion of new molecules derived from MPs and chemical libraries based on NPs in the drug discovery process is likely to increase. The renewed scientific interest and research trends in the discovery and development of MP-based NPs clearly signify that they remain a promising source of new therapeutic agents for the future. Various new methodologies coupled with technological advancements have enhanced the selection, identification, isolation, characterization, and biological screening of NPs. Progress in chromatographic and spectrometric technologies has enhanced the isolation and structural identification of bioactive compounds. System-wide approaches, such as network pharmacology, computer-aided drug discovery, pathway analysis, molecular networking and nanobiotechnology, can be employed to support NP drug discovery. Elucidating the biosynthetic pathways of NPs presents a considerable challenge, which can be addressed by various techniques, such as genetic engineering, metabolic engineering, plant cell culture, and synthetic biology. Technological advancements have made it possible to explore the profiles of intricate NPs, resulting in the isolation or synthesis of various successful therapeutic drugs and novel lead compounds that can serve as foundational structures for future medicines. Regulatory bodies across various countries must be proactive and consistently implement adequate measures to safeguard public health. It is vital that NP medicines be regulated under a drug regulatory framework to ensure compliance with established standards of safety, quality and efficacy. Despite the problems and challenges that exist, ongoing research and technological progress are anticipated to improve the effectiveness and success of drug development based on NPs. Moreover, the likelihood of developing new therapeutic agents is expected to increase. Ultimately, NPs will continue to play a vital role in drug development and our efforts to address global health challenges while also supporting the achievement of sustainable development goals related to health.

## Figures and Tables

**Table 1:** Pharmacological profile of clinically and experimentally validated plant-derived bioactive compounds.

	Drastic Phytochemical Compound	Chemical Class	Botanical Source	Key Activity: Clinical Indications/ Physiological Activity	References
1	Paclitaxel	Tetracyclic diterpenoid	*Taxus brevifolia*	Lung, ovarian, breast cancers	[57,58]
2	Artemisinin	Sequiterpene lactone	*Artemisia annua*	Malaria	[59,60]
3	Digitoxin	Cardiac glycoside	*Digitalis* spp.	Congestive heart failure, Irregular heart rhythm, Anticancer activity in lung cancer and in uveal melanoma	[61,62,63]
4	Pilocarpine	Alkaloid	*Pilocarpus jaborandi*	Stimulates saliva and sweat production, dry eye, glaucoma	[64,65,66]
5	Morphine	Opioid alkaloid	*Papaver somniferous*	Anesthetic and pain-relieving effect	[67,68]
6	Codeine	Opioid alkaloid	*Papaver somniferous*	Analgesic, antitussive	[69,70]
7	Aspirin	Acetylsalicylic acid	*Salix* spp.	Anti-inflammatory, pain-relieving, fever reduction, prevention of cardiovascular diseases, prevention of pre-eclampsia and of fetal growth restriction in pregnancy	[71,72]
8	Bifendate, (bicyclol derivative)	Lignan	*Schisandrae chinensis*	Anti-hepatitis B agent	[73,74]
9	Colchicine	Colchicinic acid	*Colchicum autumnale*	Antitumor, Gout, myocardial infarction	[75,76]
10	Camptothecin (topotecan and irinotecan analogues)	Monoterpenoid indole-alkaloid	*Camptotheca acuminata*	Anticancer	[77,78,79]
11	Vinblastine Vincristine	Vinca alkaloids	*Catharanthus roseus*	Anticancer	[80,81]
12	Galegine (derivative metformin)	Isoamylene guanidine	*Galega officinalis*	Antidiabetic action, myocardial, intestinal epithelium	[82,83]
13	Camphor	Terpenoid	*Cinnamomum camphora*	Topical analgesic, antiseptic, anti-pruritic, contraceptive, anti-inflammatory, Anticancer	[84,85]
14	Longifolene	Tricyclic sesquiterpene	*Pinus longifolia*	Antibacterial, antifungal	[86,87]
15	Delta-9-tetrahydrocan-nabinol	Cannabinoid	*Cannabis* spp.	Anti-inflammatory, post-injury pain, sleeping disorders, depression, multiple sclerosis, Nausea treatment after chemotherapy	[88,89]
16	Beta- carotene	Terpenoid (isoprenoid)	Carrots and others	Antimutagenic agents	[90,91,92,93,94]
17	Vitamin E	tocopherols, tocotrienols	Seed oils and others		
18	Ellagic acids	Polyphenol	Fruits and vegetables		
19	Organosulfuric compounds		Onion and garlic	Antibacterial activity, Antimutagenic effect, cardiovascular protective effects	[95,96,97,98,99]
20	Calanolide A	Coumarin	*Calophylium lanigerum*	Anti-HIV, Anti-tuberculosis effects	[100,101,102,103]
21	Huperzine (ZT-1 pro drug)	Alkaloid	*Huperzia serrata*	*N*-Methyl-aspartate receptor antagonist and acetylcholinesterase neuroprotective inhibitor (under trial for treatment of Alzheimer’s disease	[104,105,106]
22	Dexabinol	Cannabinoid	*Cannabis* spp.	Treatment of brain injuries (under trial, phase III)	[107,108]
23	Daidzein	Isoflavone	*Glycine max* (soybean)	Anticancer agents (under trial)	[109,110,111,112,113]
24	Protopanaxiadiol	Tetracyclic triterpene	*Panax* spp. (ginseng)		
25	Danshen	Salvianolic acid, dihydrotanshinone	*Salviae miltiorrhizae*	Treatment of diabetic retinopathy and angina pectoris	[114,115,116]
26	Borneol	Terpene derivative	*Heterothec* spp., *Artemisia* spp., *Rosmarinus officinalis* and other species		[117,118]

**Table 2:** Primary and Secondary Metabolites of Plants: Bioactive Compounds, Classification, Functions, and Mechanisms of Action.

Category	Class	Example Compounds	Function/Application	Source Plants	Mechanism of Action
Primary Metabolites	Carbohydrates	Glucose, Fructose	Energy production, structural functions	All plants	Serve as fuel in cellular respiration; precursors for cell wall components
	Proteins	Enzymes (e.g., Rubisco)	Catalyze biochemical reactions, structural support	All plants	Facilitate and regulate metabolic pathways
	Nucleic Acids	DNA, RNA	Storage and transmission of genetic information	All living cells	Genetic regulation, protein synthesis
	Lipids/Fatty Acids	Phospholipids, Oleic acid	Membrane structure, long-term energy storage	All plants	Maintain membrane integrity, participate in signaling pathways
Secondary Metabolites	Phenolics—Flavonoids	Quercetin, Luteolin, Naringenin	Antioxidant, anti-inflammatory, antiviral	Green tea, berries, soybeans	Scavenge free radicals, inhibit enzymes, block viral entry
	Phenolics—Polyphenols	Resveratrol, Catechins	Antioxidant, cardioprotective	Grapes, tea	Modulate cellular signaling pathways, inhibit oxidative stress
	Alkaloids	Quinine, Ephedrine, Homoharringtonine, Morphine	Antimalarial, decongestant, anticancer, analgesic	Cinchona, Ephedra, Cephalotaxus, Papaver somniferum	Interfere with DNA replication, modulate receptors, inhibit protein synthesis
	Terpenoids	α-Pinene, Limonene, 3-Carene, Saponins	Antimicrobial, anti-inflammatory, antifungal, insecticidal	Pine, citrus, Eremophila, Commiphora	Disrupt microbial membranes, suppress cytokine expression, block viral glycoproteins
	Essential Oils	Thymol, Menthol, Carvacrol	Antimicrobial, anti-inflammatory, aromatic	Thyme, mint, oregano	Damage bacterial cell walls, inhibit quorum sensing and biofilm formation
	Stilbenes & Lignans	Resveratrol, Pinoresinol	Antioxidant, cardioprotective	Grapes, flaxseed	Inhibit oxidative stress, modulate signaling pathways
	Isoflavones	Genistein, Daidzein	Phytoestrogenic, anticancer	Soybeans	Bind estrogen receptors, inhibit tyrosine kinases

**Table 3:** Key secondary metabolites and their biological activities covered in this review.

Metabolite Group	Plant Source	Secondary Metabolites	Activity	Reference
**Phenolics**				
Flavonols	Olive oil, onion, berries red wine, grapefruit	Quercetin, kaempferol, galangin	Antiviral, antimutagenic	[135]
Flavones	Fruit, red pepper and tomato skin, red wine	Apigenin, luteolin	Antiviral, anti-inflammatory, antimutagenic	[136]
Flavanones	Citrus fruits, grapefruits	Naringenin, hesperetin	Antibacterial, antimutagenic	[137]
Anthocyanidins	Strawberry, cherry, ruspberry	Cyanidin, delphinidine	Antioxidant	[138]
Isoflavones	Soybean	Genistein, daidzein	Antibacterial, antimutagenic	[139]
**Alkaloids**				
	*Papaver Somniferum*	Morphine	Analgetic	[140]
	*Ephedra* sp.	Ephedrine	Antiasthmatic	[141]
	*Remijia* sp.	Quinine	Antimalarial	[142]
	*Cephalotaxus fortunei*	Homoharringtonine	Anticancer	[143]
**Terpenoids**				
	Ginger, citronella, camphor, thynnus, oregano and sage essential oils	Camphone	Antibacterial, anticancer	[144]
	Coniferous trees	a-Pinene	Antimicrobial, antioxidant, anticancer, anti-inflammatory	[145]
	*Allium* species, oats, spinach, tea, asparagus	Saponins	Ant-diabetic, hypolipidemic, anticancer	[146]
	Citrus, cannabis, rosemary, basil, pine	3-Carene	Antimicrobial	[147]

**Table 4:** Medicinal plant-derived natural products with reported antimicrobial properties against drug-resistant pathogens.

Plant Species	Natural Products	Mechanism of Action	Pathogens	Reference
*Scutellariabaicalensis*	Baicalein	Efflux pump inhibition	MRSA	[162]
*Aframomumpolyanthum*	Anthocyanin Phenols Polyphenols Saponin	-	MDR *E.coli* MDR *E. aerogene* MDR *E. cloacae* MDR *K. pneumonia*	[163]
*Cinnamomum tamala*	Cinnamal dehyde	Disruption of cell membrane integrity	MDR *H. pylori*	[164]
*Aroma melanocarpa*	Ellagic acid	Inhibition of hemagglutinin protein	Oseltamivir-resistant influenza virus	[165]
*Carissa edulis*	Lupeol	-	Acyclovir-resistant HSV-1	[166]
*Mentha pulegium* L.	-	Prevention of synthesis or repression of function of alpha proteins	Acyclovir-resistant HSV-1	[167]
*Berben’s vulgaris* L.	Berberine	Increase ROS efflux transporter inhibition	Fluconazole-resistant *C. tropicalis*	[168]
*Origanum vulgare* L.	Carvacrol	Disruption of cell membrane structure–function	Azole-non susceptible *C. neoformans*	[169]
*Syzygiumaromaticum* L.	Eugenol	Disruption of cell membrane structure–function	Fluconazole-resistant *A Fumigates*	[170]

MRSA: Methicillin-resistant *Staphylococcus aureus*; MDR: Multidrug-resistant; HSV-1: Herpes simplex virus; ROS: Reactive oxygen species.

**Table 5:** Abiotic environmental factors on the concentration of various secondary metabolites.

Metabolite Group	Plant Species	Secondary Metabolite	Environmental Factor	Concentration Change	Reference
Alkaloids	*Camptotheca acuminate*	Camptophecin	27% full sunlight	Increase	[208]
Phenolics	*Vaccinium myrtillus*	Chlorogenic acid	Full sunlight	Increase	[209]
Phenolics	*Lactuca sativa*	Ferulic acid	Increase red light	Decrease	[210]
Alkaloids	*Catharanthus roseus*	Cantharantine	Ultraviolet B radiation	Increase	[211]
Alkaloids	*Papaven somniferum*	Morphine	Low temperature	Decrease	[212]
Terpenoids	*Daucus carota*	a-farnesene	High temperature	Increase	[213]
Phenolics	*Rhodiola rosea*	Salidroside	Soil moisture (55–75%)	Increase	[214]
Phenolics	*Salvia miltiorrhiza*	Tanshinone	Severe drought	Increase	[215]

**Table 6:** Standard and green technologies for extracting bioactive constituents from medicinal Plants.

Method		Principle	Reference
Standard methods	Maceration	MP is soaked in an appropriate solvent	[231,232]
	Soxhlet extraction	Continuous solvent extraction after solvent washing the MP, through a cycle of boiling and condensation	[233,234]
	Decoction	Boiling after slicing the MP	[235,236]
	Cold pressing	Mechanical force on the MP material	[237,238]
	Hydrodistilation	MPs boiled with water and after condensation the essential oils and the hydrosols are recovered separately	[239,240]
Green extraction methods (eco-extraction methods)	Superficial fluid extraction	Pressurized supercritical solvents flow through a column and dissolve extractable compounds from the solid MP material in the column	[241,242]
	Pressurized hot water extraction	Water at high temperatures shows lower polarizability/polarity and density. Its surface tension and viscosity decrease, and the diffusivity increases allowing faster mass transfer and improve wetting of the MP material.	[243,244]
	Ultrasound assisted extraction	Cavitation effect creates high pressure and high temperature zones. Also, it increases solvent penetration into the cells.	[245,246]
	Enzyme assisted extraction	Enzymes with specific hydrolytic properties are used to degrade the MP matrix to extract biodrastic components from cytosolic spaces and cell walls	[247,248]
	Microwave assisted extraction	Rapid increase of the temperature of the fresh or rehydrated MP material causes cell wall disruption and the release of chemical substances.	[249,250]
	Pulse electric field assisted extraction	If an external electric field’s strength is much higher than the critical electric value of the cell membrane, then electrical rupture occurs releasing the cell contents.	[251,252]

**Table 7:** Innovative methods for the discovery and development of pharmaceutical substances derived from plants.

Method		Application to MPs and NPs	Reference
Omics technologies	Genomics and transcriptomics	Genomics enables researchers to decode the entire genetic blueprint of medicinal plants, which is essential for identifying genes responsible for the biosynthesis of pharmacologically active compounds. Transcriptomics play a complementary role by identifying which genes are actively expressed under specific conditions, revealing biosynthetic pathways and enabling the manipulation of gene expression for enhanced yield.	[264,265,266,267]
	Metabolomics	Metabolomics refer to the large-scale study of small molecules (metabolites) within cells and tissues. With the aid of techniques such as nuclear magnetic resonance (NMR) spectroscopy and mass spectrometry (MS), researchers can study the metabolite composition of plants. This facilitates the rapid identification of bioactive compounds and their precursors,	[268,269,270,271,272]
	Proteomics	Proteomics focuses on the complete array of proteins produced by a MP. Understanding the protein expression patterns helps to identify the key enzymes as well as the regulatory proteins involved in the production of target compounds.	[273,274,275]
Artificial Intelligence and machine learning	Predicting bioactivity	AI models trained on already known bioactive compounds are able to predict the pharmacological potential of newly identified phytochemicals	[276,277,278]
	De novo compound design	Algorithms based on phytochemical molecules can generate entirely new molecular structures with desirable pharmacokinetic properties	[279,280]
	Toxicity prediction	Early identification of toxicity reduces significantly the failure rate in later development stages	[281,282]
CRISPR (Clustered Interspaced Short Palindromic Repeat) and genetic engineering	Gene-editing technologies have extended the potential of plant biotechnology	- Allow MP genome to enhance production of bioactive compounds, - Silence MP genes that divert metabolic flux away from desired products, - Introduce new biosynthetic capabilities from other organisms	[283,284,285,286,287]
Synthetic Biology and Metabolic Engineering	An application of engineering for the design and development of new biological parts, systems and devices or for the reconstruction of existing ones.	- Heterologous expression: inserting MP genes in a bacterial genome like the one of E. coli for increased yield of biomolecules. - Pathway reconstruction: Biosynthetic pathways of endangered or slow growing species of MPs can be reconstructed in bacterial cells - Optimization of protein synthesis by regulating the biosynthetic pathways with the necessary enzymes.	[288,289,290,291,292,293]
High Throughput Screening (HTS) and Automation	HTS enables the rapid evaluation of -literally-thousands of plant extracts or compounds for biological activity using automated systems.	The relevant HTS platforms integrate robotic handling, miniaturized assays, and real-time data analytics. Thus the identification of “target” candidate compounds that warrant further investigation is being significantly accelerated.	[294,295]
Nanotechnology and drug delivery innovations	It is used for formulating drugs and for screening and discovery.	Nano-sensors can detect molecular interactions at extremely low concentrations, aiding in the identification of potent phytochemicals. In the development phase, nano -formulations improve the bioavailability, solubility, and targeted delivery of plant-based drugs.	[296,297,298,299]
Chemoinformatics and Virtual screening	Facilitate the in-silico analysis of MP-derived compounds using molecular docking, quantitative structure–activity relationship (QSAR) modeling, and virtual libraries.	These methods highlight the most promising candidates before physical screening, saving time and resources	[300,301]
Ethnopharmacology (E/Ph) and Big Data	Contemporary trends combining E/Ph with informatics	When E/Ph is integrated with big data analytics, researchers can systematize and analyze traditional knowledge across cultures. Machine learning methods can analyze ethnobotanical data to predict which plants are most likely to contain bioactive compounds based on usage patterns and taxonomy.	[302,303,304]
Bioprospecting with Remote Sensing and GIS (Geographical Information Systems)	Satellite imaging and GIS permit researchers to identify and supervise biodiversity-rich regions.	Specific ecosystems or habitats may harbor novel MPs. Hence it becomes possible to predict the distribution of valuable species and plan sustainable collection strategies.	[305,306,307]

## Data Availability

Not applicable.
